# Management of an Autism Spectrum Disorder (ASD) Patient With Respiratory Failure in the Intensive Care Unit: A Case Report on the Role of Dexmedetomidine

**DOI:** 10.1155/crcc/2549155

**Published:** 2026-05-20

**Authors:** Alessandra Ciccozzi, Federico Murgia, Alba Piroli, Antonella Paladini, Claudia Ciccone, Francesca Leonardis, Davide Fionda, Antonello Ciccone, Franco Marinangeli

**Affiliations:** ^1^ Department of Life, Health and Environmental Sciences—University of L′Aquila, L′Aquila, Italy; ^2^ Department of Anaesthesia and Intensive Care, Ospedale Civile San Salvatore, University Hospital of L′Aquila, L′Aquila, Italy; ^3^ Department of Surgical Sciences, Hospital–University Company City of Health and Science of Turin, Università degli Studi di Torino Dipartimento di Scienze Chirurgiche, Turin, Italy; ^4^ Intensive Care Unit, Tor Vergata University Hospital, Università degli Studi di Roma Tor Vergata Facoltà di Medicina e Chirurgia, Rome, Italy, uniroma2.it

**Keywords:** autism spectrum disorder, dexmedetomidine, respiratory failure, sedation, special needs

## Abstract

This clinical case describes the management, in an intensive care unit (ICU), of a patient with a severe form of autism spectrum disorder (ASD) Level 3, suffering from acute respiratory failure that evolved into acute respiratory distress syndrome (ARDS). Throughout the entire period of hospitalisation in the ICU, sedation of the patient played a key role in the performance of all invasive and noninvasive therapeutic procedures, in particular artificial ventilation and subsequent weaning, due to the underlying disorder that rendered the patient completely uncooperative. Dexmedetomidine was used during initial admission to the ICU for initial treatment, during the period of artificial ventilation and finally during weaning from mechanical ventilation. The *α*_2_ agonist, due to its pharmacodynamic and pharmacokinetic characteristics, also facilitated the overlap with home antipsychotic drugs upon both discontinuation and reinstatement, allowing discharge from the ICU and safe and serene home management of the tracheostomy until its closure.

## 1. Introduction

Autism spectrum disorder (ASD) is a neurobiological developmental disorder that manifests in the first years of life and lasts throughout life. It is characterised, according to the Diagnostic and Statistical Manual of Mental Disorders, 5th edition Text Revision 2022 (DSM‐5‐TR) [1], by impaired communication and social interaction, limited interests and repetitive behaviour [2]. These patients fall into the category of patients defined in literature as ‘patients with special needs’. *Special needs* is the terminology used in clinical diagnostics and functional development to describe individuals who require assistance for disabilities that may be medical, mental or psychological [3].

These problems make the uncooperative patient difficult to manage in a hospital environment and in particular in the emergency department (ED) and ICU. In patients with ASD, in the ICU the following are essential: the need to minimise sensory stimuli (visual, auditory, olfactory, gustatory and unusual tactile) that evoke stress, and the involvement of family members in the care process to increase the patient′s comfort at all stages of hospitalisation. Among the sensory stimuli, acoustic stimuli from medical equipment (intravenous [IV] pumps, monitoring and ventilation devices), and unfamiliar voices appear to be the most involved [4]. Frequent visits by doctors can make the environment even more stressful. In addition, cognitive impairment increases the state of anxiety. Although there is a flourishing literature regarding the perioperative management of patients with special needs, there are no data on the management in the ICU of sedation, artificial ventilation and weaning of adult patients with ASD who require artificial ventilation for respiratory failure [5–11].

This clinical case describes the management of a patient suffering from a severe form of ASD Level 3 with acute respiratory failure that evolved into acute respiratory distress syndrome (ARDS).

## 2. Clinical Case

**Figure 1:**
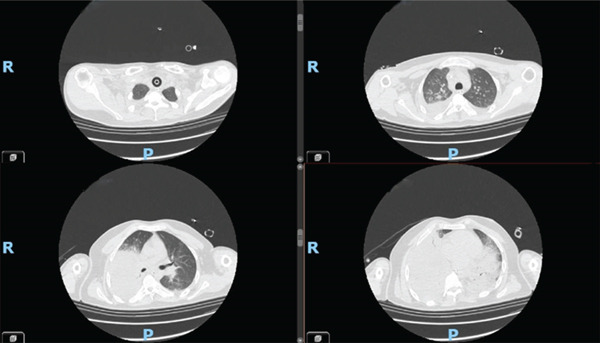
CT scan performed on admission.

**Figure 2:**
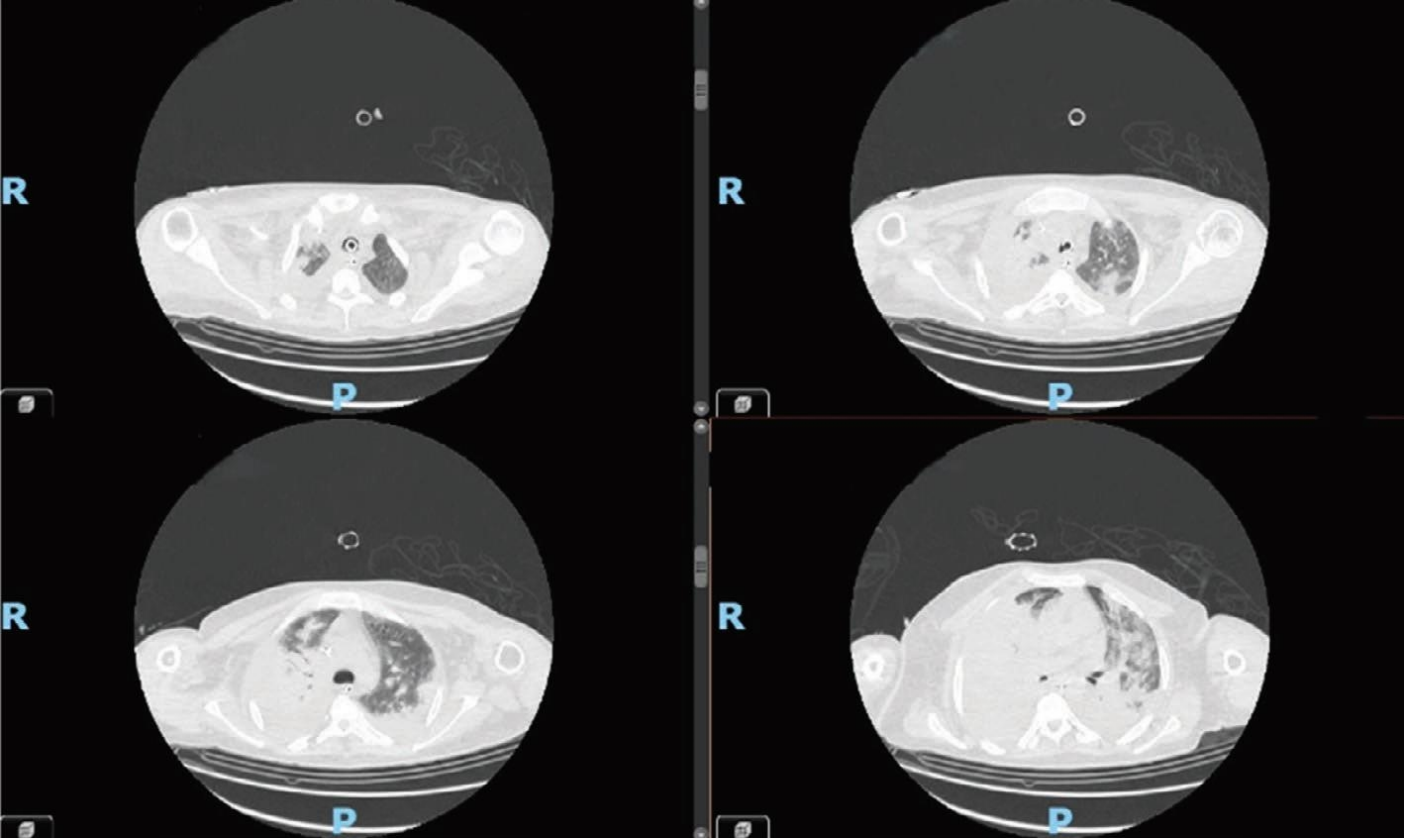
CT scan performed 10 days after admission.

A 27‐year‐old patient with severe ASD, with intellectual disability, uncooperative, arrived at the ED with worsening dyspnoea and fever. Based on the clinical picture and blood gas analysis data, a chest CT scan was performed, which documented bilateral pneumonia, involving the right lower and middle lobes and the left lower lobe and lingula, and was associated with ARDS Figures 1 and 2.

The patient′s medical history indicated, in addition to the underlying pathology, a long history of dysphagia, coeliac disease and lactose intolerance. No drug allergies were present. Home oral therapy is shown in Table 1.

**Table 1:** Home oral therapy.

Home oral therapy		
Drug	Dosage	Timetable
Paliperindone	9 mg	1 tablet in the morning
Extended‐release valproate	500 mg	1 tablet every 12 h
Extended‐release quetiapine	200 mg	1 tablet in the morning
Quetiapine	200 mg	1 tablet at 7 PM
Tavor	5 mg	1 tablet at 8 PM
Levomepromazine	100 mg	1 tablet at 8 PM

The patient had completed the vaccination cycle for SARS‐Cov2, and upon admission to the emergency room, the nasopharyngeal swab for SARS Covid‐2 was negative. The difficulty in managing an uncooperative patient was evident from the moment of admission, in performing blood gas analysis, blood chemistry tests, CT scan and in maintaining a Venturi mask for oxygen therapy. For this purpose, ketamine was administered intramuscularly at a dosage of 3 mg/kg. After admission to the ICU, a continuous IV infusion of dexmedetomidine at a dosage of 0.7 mg/kg/h and midazolam at a dosage of 0.10 mg/kg/h was started, after cardiorespiratory monitoring in order to allow oxygen therapy through high‐flow nasal cannulas (HFNC): 60 L/min with FiO_2_ of 50%. Empirical broad‐spectrum antibiotic therapy was initiated for suspected aspiration pneumonia with piperacillin–tazobactam 2 g every 8 h IV. The remaining drug therapy included pantoprazole 40 mg IV at 8:00 AM, enoxaparin sodium 6000 IU subcutaneously at 4:00 PM, furosemide 10 mg IV every 8 h. Home therapy was discontinued, with the exception of sodium valproate which was administered IV.

Screening on admission by nasal and rectal swab showed a rectal positivity to *Acinetobacter baumannii*.

At 24 h after admission, as no improvement in the clinical picture and haemogas‐analytical data was observed, and after adequate analgo‐sedation, oro‐tracheal intubation and invasive pressure‐controlled ventilation at guaranteed volume (VGRP) was performed (tidal volume [Vt] 6 mL/kg, positive end‐expiratory pressure [PEEP] 8 cmH_2_O). Inspired fraction of O_2_ (FiO_2_) 0.60, the sedation regime was maintained with Propofol in continuous IV at a dosage of 4.2 mg/kg/h and remifentanil at a dosage of 0.05 mg/kg/min, and midazolam at a dosage of 0.10 mg/kg/h. At the same time, the dexmedetomidine infusion, which had been started upon admission to the ICU was suspended. Bacteriological examination of the tracheobronchial aspirate was negative for pathogens. On Day 6, due to worsening respiratory exchanges and an increase in C‐reactive protein (CRP), linezolid was introduced, 600 mg every 12 h, IV. The following day, on Day 7, due to further worsening of respiratory exchanges (PaO_2_/FiO_2_ ratio, P/F < 150) the patient was treated with rocuronium, and muscle relaxation was maintained for 72 h. On Day 15, an improvement in PCR was observed and the decision was made to discontinue antibiotics completely.

On Day 19, a new worsening of respiratory exchanges and an increase in inflammation indices (CRP: 24.9 with an upward trend) were observed, therefore complete culture tests were performed: bronchoaspiration, urine culture and blood cultures from the central and peripheral veins. The infectious disease consultant prescribed antibiotic therapy with vancomycin 2 g/day in continuous IV infusion, cefiderocol 2 g, IV infusion, in 180 every 8 h, caspofungin, IV infusion 50 mg/day. On Day 31, antibiotic therapy was discontinued following the improvement of the clinical picture and laboratory tests, and a sedation regimen with dexmedetomidine and midazolam IV infusion was resumed. In the following days, ventilation assistance with pressure support ventilation (PSV) was established with progressive reduction of pressure support (PS) On Day 41, after withdrawal of midazolam on Day 39, the patient was extubated with a rapid shallow breathing index spontaneous breathing (RSBI) < 105 [12]. Oxygen therapy with HFNC was started again with a FiO_2_ of 60%, which was gradually reduced over the next few days. On Day 44, there was a new deterioration of respiratory exchanges (P/F < 150) with an increase in inflammation indices, for which oro‐tracheal intubation and assisted mechanical ventilation were necessary (PSV, PEEP: 8 cmH_2_O, FiO_2_: 45%). On Day 46, consent was obtained from the hitherto hesitant parents to perform a percutaneous tracheostomy. The different modes of respiratory assistance are summarised in Table 2.

**Table 2:** This table shows the duration of different modes of respiratory care during hospitalisation in the ICU.

Support device	Breathing modes	Duration
HFNC	Spontaneous	First 24 h
ETT (endotracheal tube)	Controlled mechanical ventilation	30 days
ETT	Assisted mechanical ventilation	16 days
Tracheostomy cannula	Assisted mechanical ventilation	14 days (gradual reduction of pressure support)
Tracheostomy cannula	Spontaneous with high flows	14 days
Tracheostomy cannula	Spontaneous with supplementary O_2_	31 days

The latter allowed a gradual weaning from mechanical ventilation until the restoration of spontaneous breathing with HFNC on tracheostomy (PEEP): 5 cmH_2_O, FiO_2_: 35%. On Day 78, after 14 days, the patient was placed on spontaneous breathing with O_2_ therapy, 3 L/m, until discharge from intensive care, on Day 105.

During the light sedation and complete weaning phases, wrist and ankle bandages were used as physical restraints to prevent the removal of catheters (central venous, arterial and bladder), extubation and disconnection from the ventilator, only during nighttime, when the parents were not present in the room.

Throughout the entire period of hospitalisation in the ICU, the patient′s sedation, summarised in Table 3, played a key role until day 59 when home therapy was completely resumed.

**Table 3:** This table shows the use of sedatives in the different stages of hospitalisation in the ICU.

Day	Drugs	Dosage	Notes
1	Dexmedetomidine	0.7 mg/kg/h	First 24 h: HFNC
	Midazolam	0.10 mg/kg/h	

2–30	Propofol	4.2 mg/kg/h	Mechanical ventilation
	Remifentanil	0.05 mg/kg/min	
	Midazolam	0.10 mg/kg/h	

31–46	Dexmedetomidine	0.7 mg/kg/h	Discontinuation of propofol due to increased calcium levels and of remifentanil.
	Midazolam	0.06 mg/kg/h	New sedation protocol

47–51	Dexmedetomidine	0.7 mg/kg/h	On Day 47 tracheotomy
	Midazolam	0.045 mg/kg/h	Gradual reduction of sedatives, reinstatement of antipsychotic therapy
	Home antipsychotic therapy		

51–58	Dexmedetomidine	0.5 mg/kg/h	Discontinuation of midazolam
	Home therapy		

59–104	Home therapy		Complete discontinuation of the sedation, full restoration of home therapy

105	Home therapy		Discharge from the ICU

Furthermore, the patient was admitted to a single room, which reduced the level of artificial lighting and noise from the monitoring, ventilation and infusion pump systems. Parents were allowed continuous access to the room during all daytime hours.

**Figure 3:**
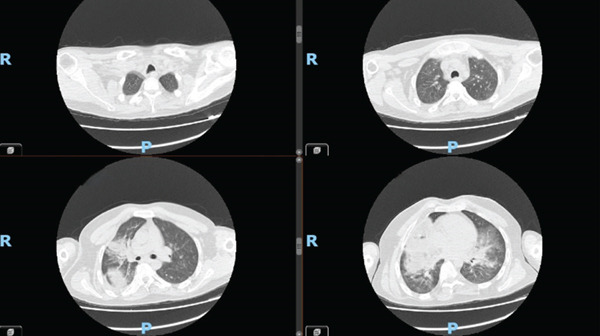
CT scan performed at discharge from ICU.

The tracheostomy cannula was removed 120 days after its placement. A CT scan performed the day after removal of the tracheostomy documented almost complete resolution of the bilateral pneumonia (Figure 3).

## 3. Discussion

In this clinical case, a key role in the management of the patient was played by pharmacological sedation, which was adequately combined with antipsychotics in home therapy. Pharmacological sedation in the ICU [13] enables the performance of invasive procedures (orotracheal intubation, central venous cannulation and tracheotomy) and daily nursing, prevents accidental extubation and ensures synchronisation between patient and ventilator during artificial ventilation [3, 4]. It also reduces sympathetic tone and oxygen consumption and indirectly stabilises the patient′s haemodynamics [14]. It controls and prevents episodes of psychomotor agitation, which are harmful to the critical patient [15, 16].

Conventional sedation combines benzodiazepines or propofol with opioids. Muscle relaxants are used primarily in the management of the acute phase of ARDS and head trauma [17–19]. During artificial ventilation, patient–ventilator interaction is influenced by the characteristics of both the ventilator and the patient′s respiratory system (respiratory rate and respiratory drive) [20, 21], which are directly affected by sedative drugs, the effects of which vary depending on the type and dose administered [22, 23]. One of the critical phases of mechanical ventilation is the weaning process from the ventilatory prosthesis. The latter requires maximum patient cooperation in order to achieve an optimal patient–ventilator interaction that balances the patient′s respiratory drive with ventilator support. An important parameter during the weaning phase, related to patient–ventilator synchronisation, is the asynchrony index (AI), calculated using the airflow curve, airway pressure and diaphragm electrical activity, which is also influenced by patient sedation [24].

The most commonly used sedatives, such as propofol, benzodiazepines and opioids, may be associated with adverse events that cause increased morbidity and prolong the clinical course of patients. They also lead to a prolongation of mechanical ventilation time, the weaning period, and consequently the length of hospital stay, with an increased risk of bacterial superinfection [25].

Patients with ASD Level 3, such as the present clinical case, require more careful pharmacological support in the ICU due to severe difficulties in understanding and communicating, both verbally and nonverbally, and the anxiety resulting from the sudden and marked change in daily routine.

Due to the severe impairment of the patient′s cognitive processes, different drugs were used for sedation purposes in the various phases, from admission to the emergency room to hospitalisation in the ICU: ketamine at the time of admission to allow the initial therapeutic and diagnostic procedures and dexmedetomidine in the first phase of hospitalisation in the ICU to allow pharmacological and respiratory therapeutic management (oxygen therapy using HFNC device); the combination of propofol–remifentanil–midazolam followed by the combination of dexmedetomidine–remifentanil–midazolam to allow the patient to adapt to the ventilator prosthesis and in general the management of the patient. Sedation in the ICU remains, therefore, a challenge for physicians and nurses with regard to intensive care, delirium prevention and clinical outcome [26].

In the present clinical case, dexmedetomidine played a fundamental role in the initial phase of admission to the ICU for primary care, during the period of artificial ventilation and finally during weaning from mechanical ventilation. It allowed an ideal level of sedation equal to the Richmond Agitation–Sedation Scale (RASS) score of −1 [18]. The *α*_2_ agonist is an increasingly popular alternative to benzodiazepines and propofol, with sedative, anxiolytic, sympathicolytic, neuroprotective and analgesic‐sparing effects with minimal respiratory depression [27–29]. Dexmedetomidine has undergone widespread clinical applications in just a few years. Procedural sedation with dexmedetomidine was, in fact, approved by the US Food and Drug Administration (FDA) in 2003, and has also been used in off‐label clinical applications: paediatric sedation, nasal and oral administration, and finally as an adjuvant to local anaesthetics [30]. It is most frequently used for paediatric sedation in noninvasive diagnostic procedures such as MRI [31]. Dexmedetomidine has important characteristics that make it particularly suitable in the ICU, providing reawakenable cooperative sedation, reducing the affective–motivational component of pain (analgognosia) and evoking indifference to the surrounding environment not associated with respiratory depression [32].

Another important aspect in the management of this patient′s drug therapy is the management of home therapy and, in particular, psychotropic medication: quetiapine, sodium valproate, lorazepam and levomepromazine. Of the latter, only sodium valproate was maintained throughout the hospital stay. Valproate, in fact, finds off‐label indication in the management of aggressive behaviour, severe irritability or mood instability.

The combination with dexmetomidine was essential when reintroducing home therapy, with gradual reduction of the *α*_2_ agonist until its suspension without any influence on the pharmacodynamics of the psychotropic drugs. No adverse events related to dexmedetomidine infusion have been reported.

Finally, the positive outcome was also achieved thanks to the involvement of family members throughout the treatment process, particularly during the dexmedetomidine de‐escalation phase. The parents′ constant presence significantly reduced the anxiety and irritability that inevitably accompany intellectual disability.

## 4. Conclusions

Sedation with dexmedetomidine, in the management of respiratory failure in a patient with a severe form of ASD, promoted the synchronisation of the patient with the ventilator prosthesis throughout the artificial support phase, particularly during weaning, due to an optimal interaction between the patient′s respiratory drive and rate and the ventilator characteristics. The *α*_2_ agonist, due to its pharmacodynamic and pharmacokinetic characteristics, facilitated the overlap with home antipsychotic drugs at the time of suspension and their reintroduction, allowing discharge from the ICU and safe and peaceful home management of the tracheostomy until its closure.

## Funding

Open access publishing facilitated by Universita degli Studi dell’Aquila, as part of the Wiley ‐ CRUI‐CARE agreement.

## Ethics Statement

Written informed consent was obtained from the patient′s guardian for the publication of this case report.

## Conflicts of Interest

The authors declare no conflicts of interest.

## Data Availability

Research data are not shared.

## References

[1] American Psychiatric Association , Diagnostic and Statistical Manual of Mental Disorders, 2022, 5th edition, American Psychiatric Association Publishing, 10.1176/appi.books.9780890425787.

[2] American Psychiatric Association , Neurodevelopmental Disorders, Diagnostic and Statistical Manual of Mental Disorders, 2022, American Psychiatric Association Publishing, 10.1176/appi.books.9780890425787.x01_Neurodevelopmental_Disorders.

[3] Wang Y.-C. , Lin I.-H. , Huang C.-H. , and Fan S. Z. , Dental Anesthesia for Patients With Special Needs, Acta Anaesthesiologica Taiwanica. (2012) 50, no. 3, 122–125, 10.1016/j.aat.2012.08.009, 2-s2.0-84871447014. 23026171

[4] Turnage D. M. and Peach B. C. , Autism in Critical Care, Critical Care Nurse. (2022) 42, no. 5, 8–10, 10.4037/ccn2022180. 36180055

[5] Okur S. , Arıkan M. , Temel G. , and Temel V. , BIS-Guided Total Intravenous Anesthesia for Orchiopexy and Circumcision in a Child With Severe Autism: A Case Report, Case Reports in Anesthesiology. (2012) 2012, 718594, 10.1155/2012/718594, 23227368. 23227368 PMC3512242

[6] Ciccozzi A. , Pizzi B. , Vittori A. , Piroli A. , Marrocco G. , Della Vecchia F. , Cascella M. , Petrucci E. , and Marinangeli F. , The Perioperative Anesthetic Management of the Pediatric Patient With Special Needs: An Overview of Literature, Children. (2022) 9, no. 10, 10.3390/children9101438, 36291372. PMC9600107 36291372

[7] Ciccozzi A. , Lupi E. , Necozione S. , Giovannetti F. , Oliva A. , Ciuffini R. , Angeletti C. , Marinangeli F. , and Piroli A. , Airway Management and General Anesthesia in Pediatric Patients With Special Needs Undergoing Dental Surgery: A Retrospective Study, Reports. (2024) 7, no. 3, 10.3390/reports7030079, 40729302. PMC12225287 40729302

[8] Thompson D. G. and Tielsch-Goddard A. , Improving Management of Patients With Autism Spectrum Disorder Having Scheduled Surgery: Optimizing Practice, Journal of Pediatric Health Care. (2014) 28, no. 5, 394–403, 10.1016/j.pedhc.2013.09.007, 2-s2.0-84906082766, 24287372. 24287372

[9] Lefevre-Scelles A. , Sciaraffa C. , Moriceau J. , Roussel M. , Croze J. , Moizan H. , Fourdrinier V. , Dureuil B. , and Compere V. , Safety of Day Surgery for Patients With Special Needs, Anaesthesia Critical Care & Pain Medicine. (2021) 40, no. 6, 100949, 10.1016/j.accpm.2021.100949. 34537388

[10] Choi J. and Doh R.-M. , Dental Treatment Under General Anesthesia for Patients With Severe Disabilities, Journal of Dental Anesthesia and Pain Medicine. (2021) 21, 87–98, 10.17245/jdapm.2021.21.2.87. 33880402 PMC8039166

[11] Marinho M. A. , Ramos F. C. T. , Cardoso A. L. , Silva-Junior G. O. , Faria M. D. B. , Bastos L. F. , Dziedzic A. , and Picciani B. L. S. , Dental Treatment Under General Anesthesia in Patients With Special Needs Provided by Private and Public Healthcare Services-a Retrospective, Comparative Study, Healthcare. (2022) 10, no. 6, 10.3390/healthcare10061147, 35742197. PMC9222989 35742197

[12] Karthika M. , Enezi F. A. A. , Pillai L. V. , and Arabi Y. M. , Rapid Shallow Breathing Index, Annals of Thoracic Medicine. (2016) 11, no. 3, 10.4103/1817-1737.176876, 2-s2.0-84979030780, 27512505. PMC4966218 27512505

[13] Sydow M. and Neumann P. , Sedation for the Critically Ill, Intensive Care Medicine. (1999) 25, no. 6, 634–636, 10.1007/s001340050917, 2-s2.0-0033144709. 10416920

[14] Nimmo G. R. , Mackenzie S. J. , and Grant I. S. , Haemodynamic and Oxygen Transport Effects of Propofol Infusion in Critically Ill Adults, Anaesthesia. (1994) 49, no. 6, 485–489, 10.1111/j.1365-2044.1994.tb03517.x, 2-s2.0-0028241922, 8017590. 8017590

[15] Kress J. P. , Pohlman A. S. , and O’Connor M. F. , Daily Interruption of Sedative Infusions in Critically Ill Patients Undergoing Mechanical Ventilation, New England Journal of Medicine. (2000) 342, no. 20, 1471–1477, 10.1056/NEJM200005183422002, 2-s2.0-0034682246, 10816184. 10816184

[16] Gehlbach B. K. and Kress J. P. , Sedation in the Intensive Care Unit, Current Opinion in Critical Care. (2002) 8, no. 4, 290–298, 10.1097/00075198-200208000-00004, 2-s2.0-0036667402. 12386488

[17] Devlin J. W. and Roberts R. J. , Pharmacology of Commonly Used Analgesics and Sedatives in the ICU: Benzodiazepines, Propofol, and Opioids, Critical Care Clinics. (2009) 25, no. 3, 431–449, 10.1016/j.ccc.2009.03.003, 2-s2.0-67649304470, 19576523. 19576523

[18] Chanques G. , Jaber S. , Jung B. , and Payen J. F. , Sédation-analgésie en réanimation de l′adulte, Encyclopedie Medico-Chirurgicale, Anesthésie Reanimation. (2013) 10, 1–12.

[19] National Heart, Lung, and Blood Institute PETAL Clinical Trials Network , Early Neuromuscular Blockade in the Acute Respiratory Distress Syndrome, New England Journal of Medicine. (2019) 380, no. 21, 1997–2008, 10.1056/NEJMoa1901686, 2-s2.0-85066092762, 31112383. 31112383 PMC6741345

[20] Thille A. W. , Rodriguez P. , Cabello B. , Lellouche F. , and Brochard L. , Patient-Ventilator Asynchrony During Assisted Mechanical Ventilation, Intensive Care Medicine. (2006) 32, no. 10, 1515–1522, 10.1007/s00134-006-0301-8, 2-s2.0-33749237011. 16896854

[21] Gilstrap D. and MacIntyre N. , Patient–Ventilator Interactions. Implications for Clinical Management, American Journal of Respiratory and Critical Care Medicine. (2013) 188, 1058–1068, 10.1164/rccm.201212-2214CI, 2-s2.0-84887295636. 24070493

[22] Goodman N. W. , Black A. M. , and Carter J. A. , Some Ventilatory Effects of Propofol as Sole Anaesthetic Agent, British Journal of Anaesthesia. (1987) 59, no. 12, 1497–1503, 10.1093/bja/59.12.1497, 2-s2.0-0023485837, 3122806. 3122806

[23] Vaschetto R. , Cammarota G. , Colombo D. , Longhini F. , Grossi F. , Giovanniello A. , Della Corte F. , and Navalesi P. , Effects of Propofol on Patient-Ventilator Synchrony and Interaction During Pressure Support Ventilation and Neurally Adjusted Ventilatory Assist, Critical Care Medicine. (2014) 42, no. 1, 74–82, 10.1097/CCM.0b013e31829e53dc, 2-s2.0-84891541056, 23982026. 23982026

[24] Conti G. , Ranieri V. M. , Costa R. , Garratt C. , Wighton A. , Spinazzola G. , Urbino R. , Mascia L. , Ferrone G. , Pohjanjousi P. , Ferreyra G. , and Antonelli M. , Effects of Dexmedetomidine and Propofol on Patient-Ventilator Interaction in Difficult-to-Wean, Mechanically Ventilated Patients: A Prospective, Open-Label, Randomised, Multicentre Study, Critical Care. (2016) 20, no. 1, 10.1186/s13054-016-1386-2, 2-s2.0-84976894788, 27368279. PMC4930611 27368279

[25] Barrientos-Vega R. , Sanchez-Soria M. M. , Morales-Garcia C. , Robas-Gomez A. , Cuena-Boy R. , and Ayensa-Rincon A. , Prolonged Sedation of Critically Ill Patients With Midazolam or Propofol: Impact on Weaning and Costs, Critical Care Medicine. (1997) 25, no. 1, 33–40, 10.1097/00003246-199701000-00009, 2-s2.0-0031012702. 8989173

[26] Page V. J. and McAuley D. F. , Sedation/Drugs Used in Intensive Care Sedation, Current Opinion in Anesthesiology. (2015) 28, no. 2, 139–144, 10.1097/ACO.0000000000000174, 2-s2.0-84926419955, 25635367. 25635367

[27] Unchiti K. , Leurcharusmee P. , Samerchua A. , Pipanmekaporn T. , Chattipakorn N. , and Chattipakorn S. C. , The Potential Role of Dexmedetomidine on Neuroprotection and Its Possible Mechanisms: Evidence From In Vitro and In Vivo Studies, European Journal of Neuroscience. (2021) 54, no. 9, 7006–7047, 10.1111/ejn.15474, 34561931. 34561931

[28] Mo Y. and Zimmermann A. E. , Role of Dexmedetomidine for the Prevention and Treatment of Delirium in Intensive Care Unit Patients, Annals of Pharmacotherapy. (2013) 47, no. 6, 869–876, 10.1345/aph.1AR708, 2-s2.0-84878519941, 23719785. 23719785

[29] Liaquat Z. , Xu X. , Zilundu P. L. M. , Fu R. , and Zhou L. , The Current Role of Dexmedetomidine as Neuroprotective Agent: An Updated Review, Brain Sciences. (2021) 11, no. 7, 10.3390/brainsci11070846, 34202110. PMC8301952 34202110

[30] Liu X. , Li Y. , Kang L. , and Wang Q. , Recent Advances in the Clinical Value and Potential of Dexmedetomidine, Journal of Inflammation Research. (2021) 14, 7507–7527, 10.2147/JIR.S346089, 35002284. 35002284 PMC8724687

[31] Mason K. P. , Zurakowski D. , Zgleszewski S. E. , Robson C. D. , Carrier M. , Hickey P. R. , and Dinardo J. A. , High Dose Dexmedetomidine as the Sole Sedative for Pediatric MRI, Pediatric Anesthesia. (2008) 18, no. 5, 403–411, 10.1111/j.1460-9592.2008.02468.x, 2-s2.0-41749089155, 18363626. 18363626

[32] Jakob S. M. , Ruokonen E. , Grounds R. M. , Sarapohja T. , Garratt C. , Pocock S. J. , Bratty J. R. , and Takala J. , Dexmedeto mi-Dine for Long-Term Sedation Investigators, Journal of Medical Sciences. (2000) 342, 1471–1477.

